# Supplementary material for: SECEDO: SNV-based subclone detection using ultra-low coverage single-cell DNA sequencing
Source: Bioinformatics. 2022 Jul 28;38(18):4293–300. doi: 10.1093/bioinformatics/btac510 (PMC9477524; doi:10.1093/bioinformatics/btac510)
Supplement: btac510_Supplementary_Data [file btac510_supplementary_data.pdf]

Supplementary Material
SECEDO: SNV-based subclone detection using ultra-low coverage single-cell DNA-sequencing

Hana Rozhoňová [1,2,3,†] , Daniel Danciu [1,4,†] , Stefan Stark [1,3,4] , Gunnar Rätsch [1,3,4,5,*] , André Kahles [1,3,4,*] , Kjong-Van Lehmann [1,3,4,6,7,*]

[1]Biomedical Informatics Group, Department of Computer Science, ETH Zurich, Zurich, Switzerland

[2]Institute of Integrative Biology, ETH Zurich, Zurich, Switzerland

[3]Swiss Institute of Bioinformatics, Lausanne, Switzerland

[4]Biomedical Informatics Research, University Hospital Zurich, Zurich, Switzerland

[5]Department of Biology, ETH Zurich, Zurich, Switzerland

[6]Cancer Research Center Cologne Essen, University Hospital Cologne, Cologne, Germany

[7]Joint Research Center for Computational Biomedicine, University Hospital RWTH Aachen, Aachen, Germany

[†] Joint first authors

[*] Joint corresponding authors

S1 Approximating the probability of the evidence

In this section we show how we approximate the probability of the evidence $P(c_1, c_2, c_3, c_4)$, where c_i represents the number of bases piled up at a certain locus, sorted by most frequent to least frequent, $\mathbf{C} = (c_1, c_2, c_3, c_4)$, $c = c_1+c_2+c_3+c_4$ is the pooled coverage at the locus, and by $C_1, \dots, C_4$ we denote the corresponding bases. Similarly, denote by $a_1, \dots, a_4$ the (unknown) counts of $C_1, \dots, C_4$ among the true alleles, from which the observations were made. For example, the true sequenced alleles might be AAACAC, so $a_1 = 4$, $a_2 = 2$, $a_3 = a_4 = 0$, but due to a sequencing error the observed bases are AAGCAC, so $c_1 = 3$, $c_2 = 2, c_3 = 1$, $c_4 = 0$ and $C_1 = A$, $C_2 = C$, $C_3 = G$, $C_4 = T$.

The goal is to approximate $P(\mathbf{C})$. This is equal to the sum over all possible (unknown) combinations of the true alleles:

$$P(\mathbf{C}) = \sum_{\mathbf{A}} P(\mathbf{C} \mid \mathbf{A})P(\mathbf{A}),$$

where $\mathbf{A} = (a_1, a_2, a_3, a_4)$. The first term quantifies the probability that sequencing a_1 bases C_1, a_2 bases C_2, etc. will result in the observation of c_1 bases C_1, c_2 bases C_2, etc., the second is the prior on the allele combination $\mathbf{A}$.

The sum above can be approximated by summing over the following four combinations of alleles:

1. All true alleles are C_1 (i.e. $a_1 = c$, $a_2 = a_3 = a_4 = 0$), i.e. all cells have the same homozygous genotype. This has prior probability p_{hom} and $P(\mathbf{C} \mid \mathbf{A})$ is equal to multinomial probability of (c_1, c_2, c_3, c_4) with probabilities $(1-\theta, \frac{\theta}{3}, \frac{\theta}{3}, \frac{\theta}{3})$, so we get:

$$P(\mathbf{C} \mid \mathbf{A}_{hom})P(\mathbf{A}_{hom}) = p_{hom}\frac{c!}{c_1!c_2!c_3!c_4!}(1-\theta)^{c_1}\left(\frac{\theta}{3}\right)^{c_2+c_3+c_4}$$

2. The locus is heterozygous C_1C_2, which has prior p_{het}. The most likely case is that in reality there were c_1 occurrences of C_1, c_2 of C_2 and then some combination of $c_3 + c_4$ of either C_1 or C_2 (the exact combination is unknown, but also irrelevant for the computation). We can then compute $P(\mathbf{C} \mid \mathbf{A}_{het})P(\mathbf{A}_{het})$ as

$$P(\mathbf{C} \mid \mathbf{A}_{het})P(\mathbf{A}_{het}) = p_{het}\frac{c!}{c_1!c_2!c_3!c_4!}\left(\frac{1}{2}-\frac{\theta}{3}\right)^{c_1+c_2}\left(\frac{\theta}{3}\right)^{c_3+c_4}$$

3. The locus was originally homozygous C_1C_1 but one haplotype suffered a somatic mutation, so the new genotype is C_1C_2 in some cells. This has prior $p_{hom}p_{mut}$. Assuming the proportion of tumor cells is 0.5, the most likely case is that in reality there were c_1 occurrences of C_1, c_2 occurrences of C_2 (about 3 times less than c_1) and then some combination $c_3 + c_4$ of either C_1 or C_2. Again, we don't know how many of the sequence errors were originally C_1 and how many C_2, and it doesn't matter. The multinomial probabilities are in this case equal to $(\frac{3}{4} - \frac{2}{3}\theta, \frac{1}{4}, \frac{\theta}{3}, \frac{\theta}{3})$ and $P(\mathbf{C} \mid \mathbf{A}_{hom,mut})P(\mathbf{A}_{hom,mut})$ can be computed as

$$P(\mathbf{C} \mid \mathbf{A}_{hom,mut})P(\mathbf{A}_{hom,mut}) = p_{hom}p_{mut}\frac{c!}{c_1!c_2!c_3!c_4!}\left(\frac{3}{4} - \frac{2\theta}{3}\right)^{c_1}\left(\frac{1}{4}\right)^{c_2}\left(\frac{\theta}{3}\right)^{c_3+c_4}$$

4. The locus was heterozygous and one somatic mutation happened, i.e. some cells have genotype C_1C_2 and some have genotype C_1C_3. This has prior $p_{het}p_{mut}$. Assuming again equally sized subclones, we have:

$$P(\mathbf{C} \mid \mathbf{A}_{\text{mut}})P(\mathbf{A}_{\text{mut}}) = p_{het}p_{mut}\frac{c!}{c_1!c_2!c_3!c_4!}\left(\frac{1}{2} - \frac{\theta}{3}\right)^{c_1}\left(\frac{1}{4}\right)^{c_2+c_3}\left(\frac{\theta}{3}\right)^{c_4}$$

We neglect all the other possibilities (e.g., the possibility of the locus undergoing more than one somatic mutation), as they have very low prior probability.

To get $P(c_1, c_2, c_3, c_4)$, we then simply sum over all four possibilities:

$$\begin{aligned}P(c_1, c_2, c_3, c_4) \approx \frac{c!}{c_1!c_2!c_3!c_4!}\Bigg[&p_{hom}(1-\theta)^{c_1}\left(\frac{\theta}{3}\right)^{c_2+c_3+c_4} + \\ &+ p_{het}\left(\frac{1}{2} - \frac{\theta}{3}\right)^{c_1+c_2}\left(\frac{\theta}{3}\right)^{c_3+c_4} + \\ &+ p_{hom}p_{mut}\left(\frac{3}{4} - \frac{2\theta}{3}\right)^{c_1}\left(\frac{1}{4}\right)^{c_2}\left(\frac{\theta}{3}\right)^{c_3+c_4} + \\ &+ p_{het}p_{mut}\frac{c!}{c_1!c_2!c_3!c_4!}\left(\frac{1}{2} - \frac{\theta}{3}\right)^{c_1}\left(\frac{1}{4}\right)^{c_2+c_3}\left(\frac{\theta}{3}\right)^{c_4}\Bigg]\end{aligned}$$

S2 Finding the optimal values of K for filtering uninformative loci

To find the optimal value of the constant K, we simulated a data set as follows: We assumed that there are only two types of cells in the sample ('healthy' and 'tumor'), that sequencing errors are unbiased and happen independently of each other with rate $\theta = 0.001$, that the somatic SNVs can only be of type AA+AB (i.e., a heterozygous mutation in tumor cells) or AB+AA (i.e., loss of heterozygosity in tumor cells) and that these two types of somatic mutations are equally likely. We also assumed that the coverage values at individual sites follow a Poisson distribution with a specified mean and that all loci are diploid. For different sample compositions (proportion of tumor cells in the sample ranging from 10 to 90%) , we simulated 10,000 results for each of the following scenarios:

- all cells have the same homozygous genotype – the counts follow a multinomial distribution with probabilities $\left(1-\theta,\ \frac{\theta}{3},\ \frac{\theta}{3},\ \frac{\theta}{3}\right)$
- a somatic mutation, healthy cells have genotype AA, tumor cells AB – the counts follow a multinomial distribution with probabilities $\left((f_h + \frac{1}{2}f_t)(1-\theta) + \frac{1}{2}\frac{\theta}{3}f_t,\ \frac{1}{2}f_t(1-\theta) + (f_h + \frac{1}{2}f_t)\frac{\theta}{3},\ \frac{\theta}{3},\ \frac{\theta}{3}\right)$
- a somatic mutation, healthy cells have genotype AB, tumor cells AA – the counts follow a multinomial distribution with probabilities $\left((f_t + \frac{1}{2}f_h)(1-\theta) + \frac{1}{2}\frac{\theta}{3}f_h,\ \frac{1}{2}f_h(1-\theta) + (f_t + \frac{1}{2}f_h)\frac{\theta}{3},\ \frac{\theta}{3},\ \frac{\theta}{3}\right)$

where f_h is the proportion of healthy cells in the sample and $f_t = 1 - f_h$ the proportion of tumor cells.

The optimal value of K was then defined as the value maximizing Youden's J [3]:

$$J = \text{sensitivity} + \text{specificity} - 1 = \frac{TP}{TP + FN} + \frac{TN}{TN + FP} - 1 = TPR - FPR,$$

where TP (true positives) are sites carrying a somatic mutation and classified as informative by our method, FN (false negatives) are sites carrying a somatic mutation and not classified as informative, TN (true negatives) are sites that do not carry a somatic mutation and are not classified as informative, FP (false positives) are sites that do not carry a somatic mutation but are classified as informative, TPR is the true positive rate, and FPR the false positive rate. The computation was done assuming both types of somatic mutations are equally likely. Note that the value of J is independent of the frequency of somatic mutations in the genome. Optimizing Youden's J consistently leads to high true positive rate (i.e., the proportion of sites carrying a somatic mutation that are classified as informative) and low false positive rate (i.e., the proportion of sites not carrying a somatic mutation that are classified as informative) (**Supplementary Figure S8**).

The optimal values of K for different combinations of pooled coverage and sample composition (proportion of healthy and tumor cells in the sample) are given in **Supplementary Table S1**. As we have no way of estimating the proportion of cells in each cluster, we used the K's from the fifth column of **Supplementary Table S1** (i.e., equivalent to two clusters of equal size) for all experiments in the manuscript. This of course leads to sub-optimal filtering performance in case the true sample composition is different, however, we have verified, using the simulated data described above, that the thresholds still work reasonably well (**Supplementary Figure S8**).

S3 Assumptions for computing the cell-to-cell similarities

We make the following simplifying assumptions:

1. The prevalence of differences between any two subclones is μ.

2. All cells are diploid.

3. The somatic SNVs are with equal probability of type AA+AB (a homozygous site in cluster 1, heterozygous in cluster 2) and AB+AA (a heterozygous site in cluster 1, homozygous in cluster 2); in particular, we do not take into account homozygous SNVs (AA+BB) or mutations that involve more than two different alleles (e.g. AB+AC) [1].

4. The mutated and germline heterozygous loci are distributed randomly and independently in the genome; in particular, they do not tend to cluster together.

5. Sequencing errors happen independently at each sequenced base with probability θ and are unbiased (i.e. all types of substitutions are equally likely).

6. We run the clustering on the set of informative positions; in the set there are loci of three types:

   - loci that carry a somatic SNV,
   - loci that have the same homozygous genotype in all cells, and were not discarded during the previous step only because of sequencing errors, and
   - loci that have the same heterozygous genotype in all cells, and were not discarded during the previous step e.g. because of preferential amplification.

   We denote the frequency of the homozygous loci in the data set by h and the frequency of the mutated loci by ϵ; the frequency of the germline heterozygous loci is then $(1 - h - \epsilon)$.

S4 Probability that two cells do not have any informative read overlaps

Let r be the average proportion of loci covered by at least one read in a single cell (for very low values of mean per-cell coverage, r is roughly equal to the coverage) and I the number of informative bases as identified by the Bayesian filtering step. The probability that a single informative locus is not covered by a read in both of two given cells is then

$$\left(1 - r^2\right)$$

and, approximating the informative loci as independent of each other, the probability that none of the informative bases is covered by a read in both cells is roughly equal to

$$\left(1 - r^2\right)^I .$$

For $r = 0.05$ and $I = 2 \cdot 10^6$ (i.e., the frequency of informative loci in the genome is roughy 1 in 16,000, as observed in our experiments), the probability is in the order of 10^{-2174}.

S5 EM-refinement of the clustering results

In this section, we use the following notation:

- C_j ... center of cluster j, $j \in \{1, \ldots, k\}$ where k is the number of clusters; an array of dimensions $I \times 4$, where I is the number of informative loci and 4 is the size of the alphabet (A, C, G, T); each element of the array lies in interval $(0, 1)$
- π_j ... prior on cluster j; the prior is proportional to the current size of the cluster
- $w_i^j = P(\text{cell } i \text{ belongs to cluster } j \mid \text{reads from cell } i, \text{ all cluster centers, all priors})$; it holds $\sum_{j=1}^{k} w_i^j = 1$
- $x_i^A[s]$ ... number of bases $\texttt{A}$ read at site s in cell i
- m ... the number of cells
- θ ... error rate

We iterate the following three steps until convergence:

1. Recompute cluster centers: First, for $j \in \{1, \ldots, k\}$ compute the unnormalized cluster center C'_j as the number of individual bases sequenced at each position, weighted by the probabilities of each cell belonging to cluster j:

$$C'_j[s, \texttt{A}] = \sum_i w_i^j x_i^A[s]$$
$$C'_j[s, \texttt{C}] = \sum_i w_i^j x_i^C[s]$$
$$\vdots$$

then normalize:

$$C_j[s, \texttt{A}] = \begin{cases} C'_j[s, \texttt{A}] \frac{1-(\chi_0)_j[s]\cdot\theta}{\sum_{B\in\{\texttt{A},\texttt{C},\texttt{G},\texttt{T}\}} C'_j[s,B]} & \text{if } C'_j[s, \texttt{A}] > 0 \\ \theta & \text{if } C'_j[s, \texttt{A}] = 0 \end{cases}$$
$$C_j[s, \texttt{C}] = \begin{cases} C'_j[s, \texttt{C}] \frac{1-(\chi_0)_j[s]\cdot\theta}{\sum_{B\in\{\texttt{A},\texttt{C},\texttt{G},\texttt{T}\}} C'_j[s,B]} & \text{if } C'_j[s, \texttt{C}] > 0 \\ \theta & \text{if } C'_j[s, \texttt{C}] = 0, \end{cases}$$
$$\vdots$$

where $(\chi_0)_j[s]$ is the number of elements of $C'_j[s, \bullet]$ equal to zero.

2. Recompute the weights: For each cell i and each cluster j, compute

$$\begin{aligned} w'^j_i &= P(\text{cell } i \text{ belongs to cluster } j \mid \text{reads from cell } i, \text{ all cluster centers, all priors}) \\ &\propto \pi_j \cdot P(\text{reads from cell } i \mid C_j) \\ &= \pi_j \prod_{s=1}^{I} (C_j[s, \texttt{A}])^{x_i^A[s]} \ldots (C_j[s, \texttt{T}])^{x_i^T[s]} . \end{aligned}$$

Normalize so that it holds $\sum_{j=1}^{k} w_i^j = 1$.

3. Recompute priors:

$$\pi_j = \frac{\sum_i w_i^j}{m}$$

To say that the process has converged, we require that the change in w_i^j's is smaller than 10^{-2} for all w_i^j.

To obtain the final assignments, we assign cell i to cluster j if it holds that $w_i^j \geq 0.95$, i.e. the cell belongs to cluster j with at least 95 % probability. If no such cluster exists, the cell is discarded.

S6 Variant calling: the MAQ approach

In this section we describe a simple variant calling approach inspired by the MAQ algorithm [2]. We start by creating a pseudo-bulk sample by pooling together data from all cells in one cluster. Then we call the most likely genotype for each cluster separately.

More precisely, for each site s with sufficient coverage (see below), we calculate the posterior distribution of all possible genotypes and call the most likely genotype:

$$G_j[s] = \max_{g \in \mathcal{G}} P\left(g \mid x_j^A[s], x_j^C[s], x_j^G[s], x_j^T[s], \theta\right), \tag{S1}$$

where by $\mathcal{G}$ we denote the set of all possible genotypes (AA, AC, AG, AT, CC, etc.), by $x_j^A[s], x_j^C[s], x_j^G[s]$ and $x_j^T[s]$ the number of A's, C's, G's and T's, resp., read at position s in cluster j, and by θ we denote the error rate. Using Bayes theorem we can rewrite **Equation S1** as

$$\begin{aligned} G_j[s] &= \max_{g \in \mathcal{G}} \frac{P\left(x_j^A[s], x_j^C[s], x_j^G[s], x_j^T[s] \mid g, \theta\right) P(g)}{P\left(x_j^A[s], x_j^C[s], x_j^G[s], x_j^T[s] \mid \theta\right)} \\ &= \max_{g \in \mathcal{G}} P\left(x_j^A[s], x_j^C[s], x_j^G[s], x_j^T[s] \mid g, \theta\right) P(g). \end{aligned} \tag{S2}$$

The probability of $x_j^A[s], x_j^C[s], x_j^G[s], x_j^T[s]$ given genotype g and error rate θ follows a multinomial distribution with probabilities $\left(1-\theta, \frac{\theta}{3}, \frac{\theta}{3}, \frac{\theta}{3}\right)$ if g is homozygous and $\left(\frac{1}{2}-\frac{\theta}{3}, \frac{1}{2}-\frac{\theta}{3}, \frac{\theta}{3}, \frac{\theta}{3}\right)$ if g is heterozygous. The priors are 1 if g is homozygous and 0.001 if g is heterozygous, as chosen by the authors of MAQ [2].

Notice that the genotype maximizing **Equation S1** does not need to be unique. If such a situation happens, we do not call any genotype for the given site.

Variant calling is then simply done by comparing the genotypes called in individual clusters.

To decrease the number of false calls, we further only call genotypes for sites with coverage at least 9 (in the given cluster) because at low-coverage sites there is a high chance of an incorrect genotype call. In particular, if the coverage of a site is smaller or equal to 3 and assuming $\theta = 0.001$, it is actually impossible to call a heterozygous genotype using the algorithm described above. Similarly, if the site is covered by 4 reads, the probability of correctly calling a heterozygous genotype is only 37.4%. Hence, we decided to call genotypes only for sites where the coverage is large enough, such that the theoretical chance of correctly calling both homozygous and heterozygous genotype is at least 95%.

We denote coverage by c (meant is the within-cluster pooled coverage) and sequencing error rate by θ, $\theta = 0.001$. We assume all cells are assigned to the correct cluster, i.e. all cells within one cluster have the same genotype. We want to find minimum c such that it holds both

$$P(\text{correct genotype call} \mid c, \text{true genotype homozygous}) \geq 0.95$$

and

$$P(\text{correct genotype call} \mid c, \text{true genotype heterozygous}) \geq 0.95.$$

(By 'correct genotype call' we mean here and everywhere below 'correct and unique'.) Notice that

$$\begin{aligned} &P(\text{correct genotype call} \mid c, \text{true genotype homozygous}) \\ &\quad = \sum_{B \in \{A,C,G,T\}} P(\text{correct genotype call} \mid c, \text{true genotype is } BB) \cdot P(BB \mid \text{true genotype homozygous}) \\ &\quad = P(\text{correct genotype call} \mid c, \text{true genotype is } XX), \end{aligned} \tag{S3}$$

for any $X \in \{\text{A, C, G, T}\}$, since we assume uniform prior on all homozygous genotypes and non-biasness of sequencing errors. Similarly,

$$P(\text{correct genotype call} \mid c, \text{true genotype heterozygous}) = P(\text{correct genotype call} \mid c, \text{true genotype is } XY) \tag{S4}$$

for any $X, Y \in \{\text{A,C,G,T}\}, X \neq Y$.

Equation S3 can be rewritten as a sum over all possible results $\mathcal{D}$, where a 'result' is an ordered quadruple (x_A, x_C, x_G, x_T), giving the number of sequenced A's, C's, G's and T's at the chosen locus:

$$P(\text{correct genotype call} \mid c, \text{true genotype is } XX) = \sum_{d \in \mathcal{D}} P(d \mid c, \text{true genotype is } XX)\mathbb{I}(\text{correct genotype call} \mid d), \tag{S5}$$

where by $\mathbb{I}(\cdot)$ we denote the indicator function. Similarly

$$P(\text{correct genotype call} \mid c, \text{true genotype is } XY) = \sum_{d \in \mathcal{D}} P(d \mid c, \text{true genotype is } XY)\, \mathbb{I}\left(\text{correct genotype call} \mid d\right). \tag{S6}$$

The probability of a particular result d given the true genotypes follows a multinomial distribution with probabilities $p = \left(1 - \theta, \frac{\theta}{3}, \frac{\theta}{3}, \frac{\theta}{3}\right)$ (up to ordering) in case of a homozygous genotype, and $p = \left(\frac{1}{2} - \frac{\theta}{3}, \frac{1}{2} - \frac{\theta}{3}, \frac{\theta}{3}, \frac{\theta}{3}\right)$ in case of a heterozygous genotype (again up to ordering).

We numerically computed the probabilities given by **Equation S5** and **Equation S6** for coverage ranging from 1 to 20, always enumerating all possible results. The resulting probabilities for homozygous and heterozygous genotypes are plotted in **Supplementary Figure S9**. The smallest c such that both the probabilities are at least 95%, is $c = 9$.

References

[1] Kristian Cibulskis et al. "Sensitive detection of somatic point mutations in impure and heterogeneous cancer samples". In: *Nature Biotechnology* 31.3 (2013), pp. 213–219. DOI: 10.1038/nbt.2514.

[2] H. Li, J. Ruan, and R. Durbin. "Mapping short DNA sequencing reads and calling variants using mapping quality scores". In: *Genome Research* 18 (2008), pp. 1851–1858.

[3] W. J. Youden. "Index for rating diagnostic tests". In: *Cancer* 3.1 (1950), pp. 32–35.

Supplementary tables

Table S1: Optimal values for the pre-processing threshold $\log(K)$, for different combinations of mean pooled coverage and sample composition. The simulation was performed as described in **Supplemental Material S2**, the coverage was sampled independently for each site from a Poisson distribution with the specified mean.

		Proportion of tumour cells								
		0.1	0.2	0.3	0.4	0.5	0.6	0.7	0.8	0.9
Mean pooled coverage	10	-1.729	-1.709	-1.561	-1.561	-1.601	-1.561	-1.709	-1.561	-1.729
	20	-1.390	-1.388	-1.390	-1.394	-1.394	-1.394	-1.389	-1.389	-1.390
	30	-1.386	-1.386	-1.386	-1.386	-1.386	-1.386	-1.386	-1.386	-1.386
	40	-1.386	-1.386	-1.386	-1.386	-1.386	-1.386	-1.386	-1.386	-1.386
	50	-1.386	-1.386	-1.386	-1.386	-1.386	-1.386	-1.386	-1.386	-1.386
	60	-1.386	-1.386	-1.386	-1.386	-1.399	-1.386	-1.386	-1.386	-1.386
	70	-1.386	-1.386	-1.386	-1.388	-3.673	-1.389	-1.386	-1.386	-1.386
	80	-1.386	-1.386	-1.386	-1.388	-11.48	-1.389	-1.388	-1.386	-1.386
	90	-1.386	-1.386	-1.388	-11.482	-27.492	-11.481	-1.388	-1.386	-1.386
	100	-1.386	-1.386	-1.388	-27.492	-43.502	-3.592	-1.388	-1.386	-1.386
	110	-1.386	-1.388	-3.592	-19.486	-43.502	-27.492	-1.388	-1.388	-1.386
	120	-1.386	-1.388	-11.481	-27.492	-75.524	-35.497	-11.481	-1.388	-1.386
	130	-1.386	-1.388	-19.486	-51.508	-59.513	-43.502	-3.592	-1.388	-1.386
	140	-1.386	-1.388	-11.481	-59.513	-83.529	-51.508	-11.481	-1.388	-1.386
	150	-1.386	-1.388	-35.497	-75.524	-91.535	-51.508	-27.492	-1.388	-1.386
	160	-1.386	-3.592	-35.497	-67.519	-123.556	-67.519	-43.502	-3.592	-1.386
	170	-1.386	-3.592	-35.497	-91.535	-131.562	-99.540	-43.502	-3.592	-1.388
	180	-1.388	-11.481	-59.513	-115.551	-139.56	-99.540	-35.497	-11.481	-1.388
	190	-1.388	-11.481	-67.519	-123.55	-163.583	-107.54	-59.513	-19.486	-1.388
	200	-1.388	-19.486	-51.508	-107.545	-163.583	-107.545	-75.524	-3.592	-1.388

Table S2: Precision and recall for the variants detected by SECEDO in each subclone of the synthetic dataset in **Figure 3** using a simple MAQ-based approach [2].

Subclone	Recall	Precision
Tumor1	0.92	0.36
Tumor2	0.91	0.43
Tumor3	0.92	0.46
Tumor4	0.91	0.53
Tumor5	0.92	0.67
Tumor6	0.92	0.51
Tumor7	0.92	0.53
Tumor8	0.91	0.68

Table S3: Precision and recall for each of the clusters detected by SECEDO on the synthetic dataset in **Figure 3** for various parameter settings: h, the fraction of the homozygous loci in the set of selected positions, ϵ, the fraction of the mutated loci in the set, and θ, the error rate. The parameter set used in our experiments is marked as bold.

Params	Healthy	Tumor1	Tumor2	Tumor3	Tumor4	Tumor5	Tumor6	Tumor7	Tumor8
$h = 0.3, \theta = 0.005, \epsilon = 0.01$	1.00 0.50	0.97 0.98	1.00 1.00	1.00 1.00	1.00 1.00	0.96 0.96	1.00 1.00	0.99 0.98	0.96 0.96
$h = 0.3, \theta = 0.005, \epsilon = 0.05$	1.00 0.97	0.87 1.00	1.00 1.00	1.00 0.97	1.00 0.98	0.94 0.96	1.00 0.97	1.00 0.99	0.96 0.98
$h = 0.3, \theta = 0.005, \epsilon = 0.1$	1.00 1.00	1.00 0.97	1.00 1.00	1.00 1.00	1.00 1.00	0.98 0.99	1.00 1.00	0.98 1.00	0.99 0.98
$h = 0.3, \theta = 0.005, \epsilon = 0.2$	1.00 1.00	0.99 0.99	1.00 1.00	1.00 1.00	1.00 1.00	0.99 0.99	1.00 1.00	1.00 0.99	0.99 0.99
$h = 0.3, \theta = 0.01, \epsilon = 0.01$	1.00 1.00	0.98 0.99	1.00 1.00	1.00 1.00	1.00 1.00	0.98 0.98	1.00 0.99	0.99 0.99	0.98 0.98
$h = 0.3, \theta = 0.01, \epsilon = 0.05$	1.00 1.00	0.99 0.99	1.00 1.00	1.00 0.99	1.00 1.00	0.98 0.98	1.00 1.00	0.99 0.99	0.98 0.98
$h = 0.3, \theta = 0.01, \epsilon = 0.1$	1.00 1.00	0.99 0.99	1.00 1.00	1.00 1.00	1.00 1.00	0.98 0.99	1.00 1.00	1.00 0.99	0.99 0.98
$h = 0.3, \theta = 0.01, \epsilon = 0.2$	1.00 1.00	0.99 1.00	1.00 1.00	1.00 1.00	1.00 1.00	0.99 0.99	1.00 1.00	1.00 1.00	0.99 0.99
$h = 0.3, \theta = 0.05, \epsilon = 0.01$	1.00 1.00	0.99 1.00	1.00 1.00	1.00 1.00	1.00 1.00	0.99 0.99	1.00 1.00	1.00 0.99	0.99 0.98
$h = 0.3, \theta = 0.05, \epsilon = 0.05$	1.00 1.00	0.99 1.00	1.00 1.00	1.00 1.00	1.00 1.00	0.99 0.99	1.00 1.00	1.00 0.99	0.99 0.99
$h = 0.3, \theta = 0.05, \epsilon = 0.1$	1.00 1.00	0.42 0.54	0.99 0.54	1.00 1.00	1.00 1.00	0.99 0.99	1.00 1.00	0.78 0.51	0.99 0.99
$h = 0.3, \theta = 0.05, \epsilon = 0.2$	1.00 1.00	1.00 0.94	1.00 1.00	0.99 1.00	1.00 1.00	0.99 0.99	1.00 1.00	0.97 0.51	0.99 0.99
$h = 0.5, \theta = 0.005, \epsilon = 0.01$	1.00 0.99	0.94 0.99	1.00 1.00	0.98 1.00	1.00 1.00	0.99 0.99	1.00 1.00	1.00 0.99	0.99 0.99
$h = 0.5, \theta = 0.005, \epsilon = 0.1$	1.00 1.00	1.00 0.99	1.00 1.00	1.00 1.00	1.00 1.00	0.99 0.99	1.00 1.00	1.00 1.00	0.99 0.99
$h = 0.5, \theta = 0.005, \epsilon = 0.2$	1.00 1.00	1.00 0.99	1.00 1.00	0.99 1.00	1.00 1.00	0.99 0.99	1.00 0.99	1.00 1.00	0.99 0.99
$h = 0.5, \theta = 0.01, \epsilon = 0.01$	1.00 1.00	0.99 1.00	1.00 1.00	1.00 1.00	1.00 1.00	0.99 0.99	1.00 1.00	1.00 1.00	0.99 0.98
$\boldsymbol{h = 0.5, \theta = 0.01, \epsilon = 0.05}$	1.00 1.00	1.00 0.99	1.00 1.00	1.00 1.00	1.00 1.00	0.99 0.99	1.00 1.00	1.00 1.00	0.99 0.99
$h = 0.5, \theta = 0.01, \epsilon = 0.1$	1.00 1.00	1.00 0.99	1.00 1.00	1.00 1.00	1.00 1.00	0.99 0.99	1.00 1.00	1.00 1.00	0.99 0.99
$h = 0.5, \theta = 0.01, \epsilon = 0.2$	1.00 1.00	1.00 1.00	1.00 1.00	1.00 1.00	1.00 1.00	0.99 0.99	1.00 1.00	1.00 1.00	0.99 0.99
$h = 0.5, \theta = 0.05, \epsilon = 0.01$	1.00 1.00	1.00 1.00	1.00 1.00	1.00 1.00	1.00 1.00	0.99 0.99	1.00 1.00	1.00 1.00	0.99 0.99
$h = 0.5, \theta = 0.05, \epsilon = 0.05$	1.00 1.00	1.00 1.00	1.00 1.00	1.00 1.00	1.00 1.00	0.99 0.99	1.00 1.00	1.00 1.00	0.99 0.99
$h = 0.5, \theta = 0.05, \epsilon = 0.1$	1.00 1.00	1.00 1.00	1.00 1.00	1.00 1.00	1.00 1.00	0.99 0.99	1.00 1.00	1.00 1.00	0.99 0.99
$h = 0.5, \theta = 0.05, \epsilon = 0.2$	1.00 1.00	1.00 1.00	1.00 1.00	1.00 1.00	1.00 1.00	0.99 0.99	1.00 1.00	1.00 1.00	0.99 0.99
$h = 0.8, \theta = 0.005, \epsilon = 0.01$	1.00 1.00	0.99 0.99	1.00 1.00	0.99 1.00	1.00 1.00	0.98 0.98	1.00 0.99	0.99 0.99	0.98 0.98
$h = 0.8, \theta = 0.005, \epsilon = 0.05$	1.00 1.00	0.99 0.99	1.00 1.00	0.99 1.00	1.00 1.00	0.98 0.98	1.00 0.99	0.99 0.99	0.98 0.98
$h = 0.8, \theta = 0.005, \epsilon = 0.1$	1.00 1.00	0.99 0.99	1.00 1.00	0.99 1.00	1.00 1.00	0.98 0.98	1.00 0.99	0.99 0.99	0.98 0.98
$h = 0.8, \theta = 0.005, \epsilon = 0.2$	1.00 1.00	0.99 0.99	1.00 1.00	0.99 1.00	1.00 1.00	0.98 0.98	1.00 0.99	0.99 0.99	0.98 0.98
$h = 0.8, \theta = 0.01, \epsilon = 0.01$	1.00 1.00	0.99 0.99	1.00 1.00	0.99 1.00	1.00 1.00	0.98 0.98	1.00 0.99	0.99 0.99	0.98 0.98
$h = 0.8, \theta = 0.01, \epsilon = 0.05$	1.00 1.00	0.99 0.99	1.00 1.00	0.99 1.00	1.00 1.00	0.98 0.98	1.00 0.99	0.99 0.99	0.98 0.98
$h = 0.8, \theta = 0.01, \epsilon = 0.1$	1.00 1.00	0.99 0.99	1.00 1.00	0.99 1.00	1.00 1.00	0.98 0.98	1.00 0.99	1.00 0.99	0.98 0.98
$h = 0.8, \theta = 0.01, \epsilon = 0.2$	1.00 1.00	0.99 0.99	1.00 1.00	0.99 1.00	1.00 1.00	0.99 0.98	1.00 0.99	0.99 0.99	0.99 0.99
$h = 0.8, \theta = 0.05, \epsilon = 0.01$	1.00 1.00	1.00 1.00	1.00 1.00	0.99 1.00	1.00 1.00	0.99 0.99	1.00 0.99	1.00 1.00	0.99 0.99
$h = 0.8, \theta = 0.05, \epsilon = 0.05$	1.00 1.00	1.00 1.00	1.00 1.00	0.99 1.00	1.00 1.00	0.99 0.99	1.00 1.00	1.00 1.00	0.99 0.99
$h = 0.8, \theta = 0.05, \epsilon = 0.1$	1.00 1.00	1.00 1.00	1.00 1.00	0.99 1.00	1.00 1.00	0.99 0.99	1.00 0.99	1.00 1.00	0.99 0.99
$h = 0.8, \theta = 0.05, \epsilon = 0.2$	1.00 1.00	0.99 0.99	1.00 1.00	1.00 0.99	1.00 1.00	0.98 0.99	1.00 0.99	1.00 1.00	0.99 0.98

Supplementary figures

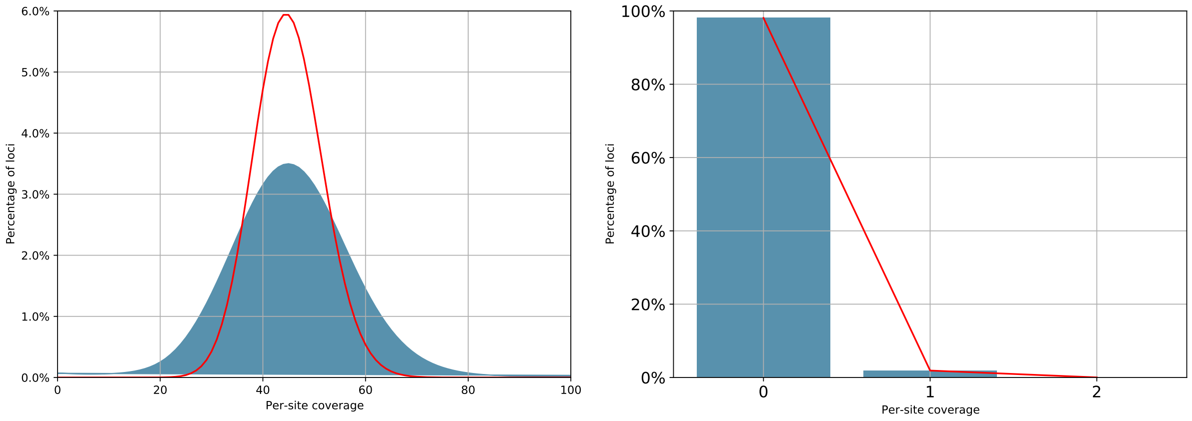

Figure S1: Coverage for Slice B of the breast cancer dataset. Left: Histogram of the per-site coverage pooled over all cells (in blue) and the theoretical Poisson distribution with the same mean (red line) Right: Histogram of the per-site coverage for individual cells. The red line is the theoretical Poisson fit.

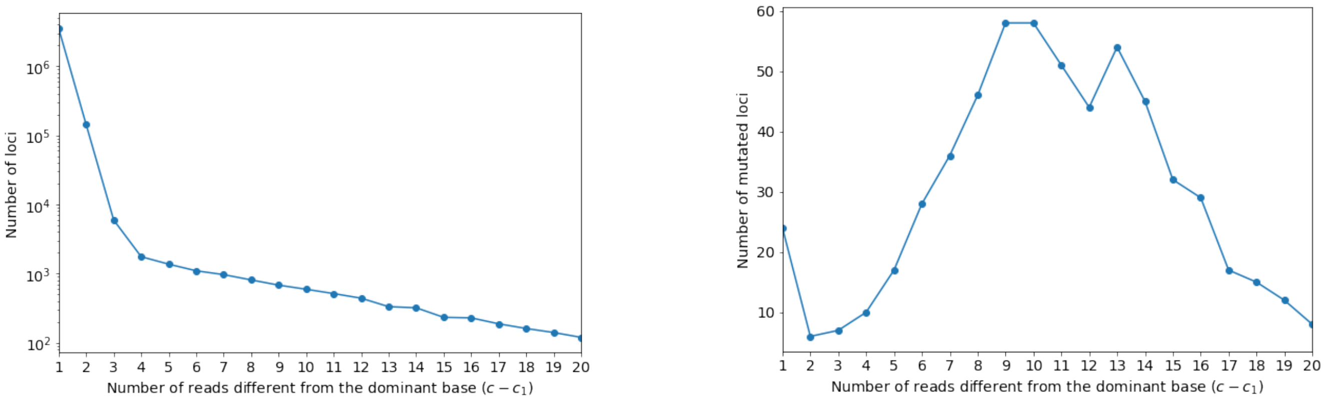

Figure S2: **Left:** Total number of loci for a given value of $c-c_1$, i.e. the number of bases different from the dominant base (log scale). **Right:** Number of mutated loci for increasing values of $c - c_1$. The values were computed from all loci of Chromosome 8 of the synthetic data set in **Figure 3**. SECEDO eliminates all loci with $c - c_1 < 5$, thus removing 99.7% of the loci while keeping 95% of the mutated loci.

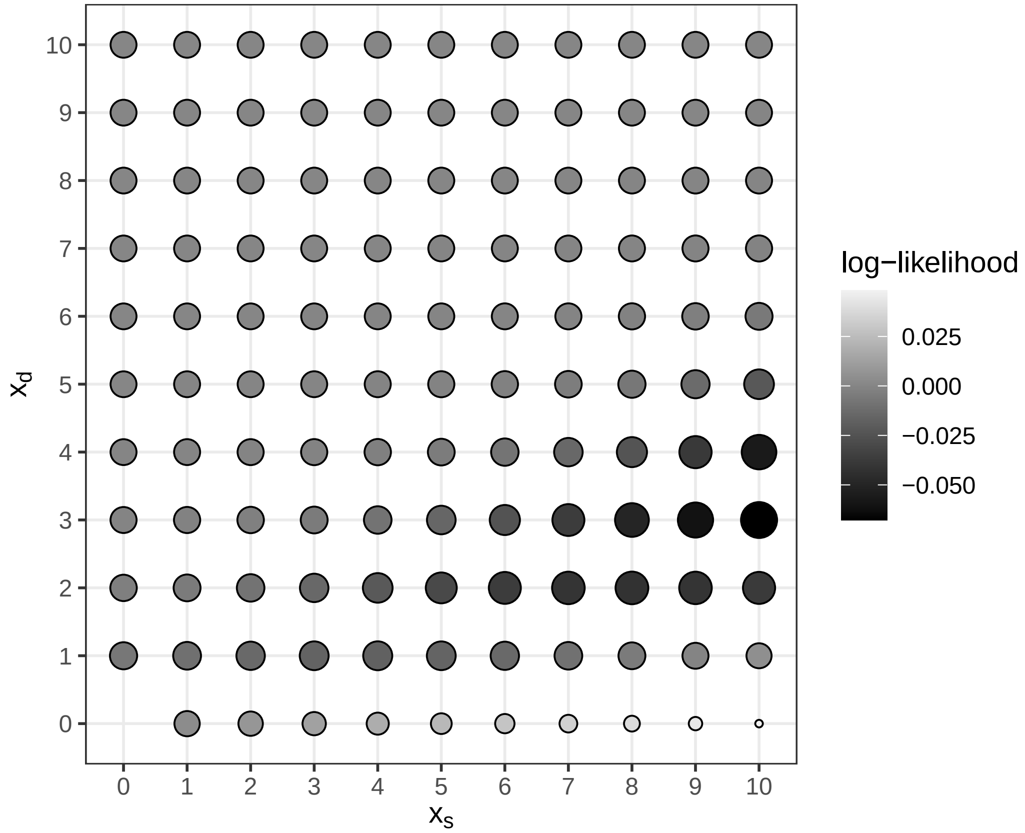

Figure S3: Informativeness of different combinations of number of matches (x_s) and mismatches (x_d) in a read overlap, computed as $\frac{P[x_s,x_d|C(i)=C(j),h,\epsilon]}{P[x_s,x_d|C(i)\neq C(j),h,\epsilon]}$. Darker and larger points denote more informative combinations. Computed using $\theta = 0.05$, $\epsilon = 0.01$, $h = 0.5$ (the same values of parameters as used for the analyses of the simulated and real data sets in the manuscript).

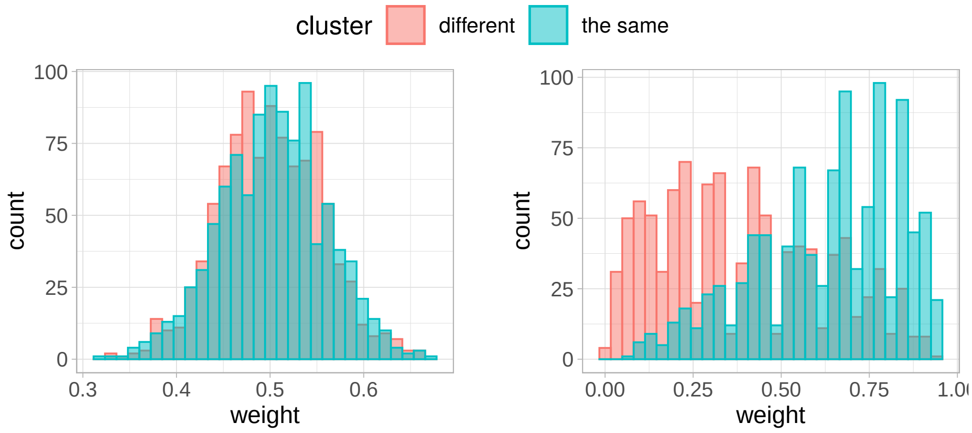

(a) Each locus treated independently (b) Overlap structure taken into account

Figure S4: Considering the overlaps as a whole provides more information than considering each locus independently. In each simulation, we simulated 550 read overlaps of length 2, assuming $\epsilon = 10^{-2}$, $h = 0$, $\theta = 10^{-3}$. The resulting weight, computed as $\frac{P[x_s,x_d|C(i)=C(j),h,\epsilon]}{P[x_s,x_d|C(i)\neq C(j),h,\epsilon]}$, was computed treating each locus independently (a) or considering the whole overlaps (b). In both cases there were 1000 simulations. The simulated data used in (a) and (b) were the same.

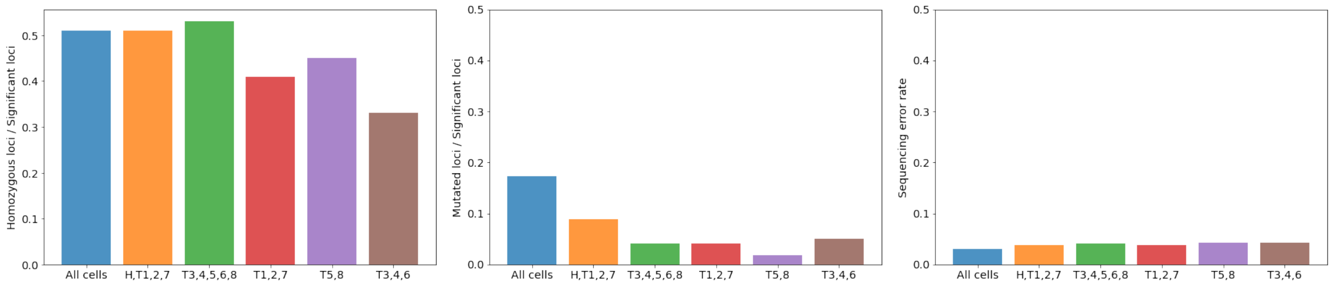

Figure S5: Estimating h, ϵ, θ based on synthetic data. **Left:** The first chart depicts the ratio of homozygous loci in the positions that pass the SECEDO filtering step at each clustering node of the simulated dataset. The ratio of homozygous loci decreases with the number of subclones (and thus with coverage), as sequencing errors are distributed among the subclones, while real mutations are kept together. **Middle:** Ratio of mutated loci as given by the ground truth VCF files generated by Varsim. **Right:** Estimated sequencing error rate by processing 1000 random homozygous loci that passed SECEDO's filtering step.
As a general rule, it is recommended to choose parameters close to the values estimated for the last level of clustering (the last three bars) as at this point the coverage is lower and SECEDO is more sensitive to parameter choices (a high coverage provides more information for each locus and can thus mitigate incorrect parameter choices).

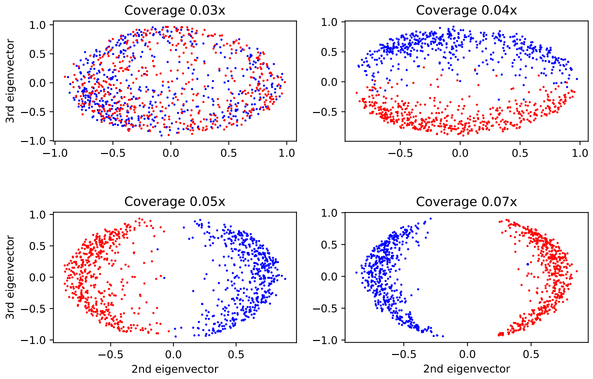

Figure S6: Scatter plots of the 2nd and 3rd eigenvectors of the similarity matrix Laplacian for increasing coverage values (synthetic data with 1,000 cells, 500 in each group; 5,000 SNVs). The higher the coverage, the clearer the separation between the two clusters.

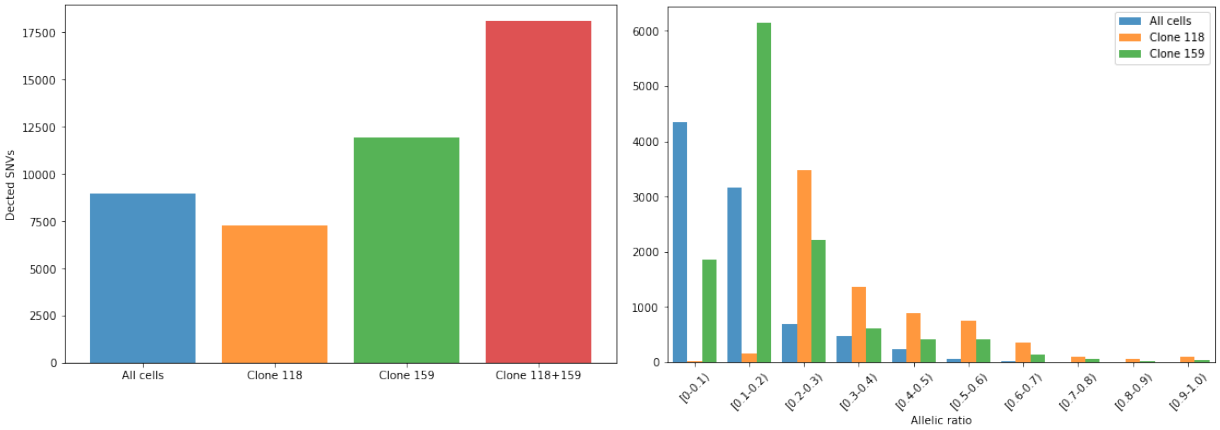

Figure S7: **Left:** SNVs detected on Slice B of the breast cancer dataset by running Mutect with the default settings. The middle columns show the number of SNVs reported when running Mutect on each of the two cancer subclones separately, as detected by SECEDO. The last column shows the number of SNVs reported by Mutect when merging the two cancer subclones, while the significantly lower value in the first column represents the number of SNVs reported by Mutect when run against all cells in the tumor slice (including the healthy cells). **Right:** Histogram of the allelic ratio for the SNVs detected by applying MuTect to the two tumor subclones and to all cells in slice B of the breast cancer dataset. The shift to the right in the allelic ratio indicates that the clustering increased the tumor purity.

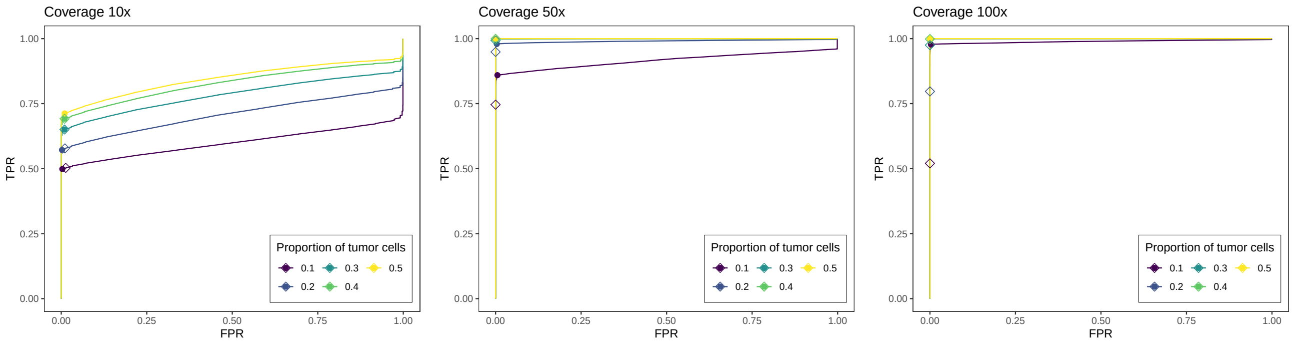

Figure S8: ROC curves for the identification of loci carrying a somatic mutation, for different values of mean pooled coverage. The simulation was done as described in **Section S2**. The full circles denote the TPR and FPR for the optimal value of K, for a given coverage and tumor proportion, as chosen by optimizing Youden's J. The empty diamonds denote the TPR and FPR obtained when using the value of K corresponding to a given coverage and tumor proportion 0.5. For clarity, only the curves for tumor proportions 0.1 to 0.5 are shown. TPR = true positive rate, FPR = false positive rate.

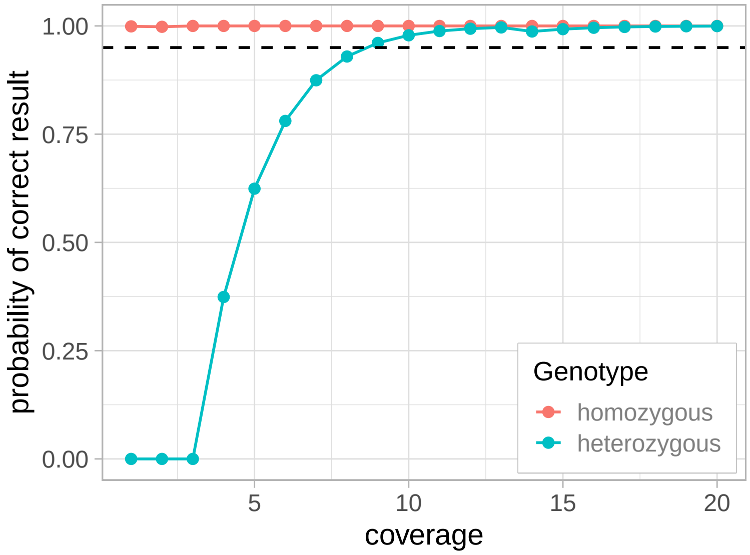

Figure S9: Probability of calling a correct and unique genotype for different values of the (within-cluster) coverage, assuming the true genotype is homozygous or heterozygous. The dashed line denotes probability 95%. See **Supplemental Material S6** for details.
